# Individualized Web-Based Attention Training With Evidence-Based Counseling to Address HIV Treatment Adherence and Psychological Distress: Exploratory Cohort Study

**DOI:** 10.2196/18328

**Published:** 2021-01-28

**Authors:** Eric Houston, Javad Salehi Fadardi, Nina T Harawa, Chris Argueta, Sukrit Mukherjee

**Affiliations:** 1 Claremont Graduate University Claremont, CA United States; 2 David Geffen School of Medicine University of California, Los Angeles Los Angeles, CA United States; 3 Charles R Drew University of Medicine and Science Los Angeles, CA United States; 4 California Pacific Medical Center Research Institute San Francisco, CA United States

**Keywords:** depression, trauma, HIV, attention training, implicit cognition

## Abstract

**Background:**

The prevalence of mood, trauma, and stressor-related disorders is disproportionately higher among people living with HIV than among individuals without the virus. Poor adherence to HIV treatment and heightened psychological distress have been linked to symptoms associated with these disorders.

**Objective:**

The objective of this exploratory pilot study was to develop and implement an intervention that combined individualized web-based attention training with evidence-based counseling to promote HIV treatment adherence and reduce psychological distress among people living with HIV. The study targeted African American and Latino young men who have sex with men, two population groups in the US that continue to experience disparities in HIV treatment outcomes.

**Methods:**

Study participants with elevated symptoms of depression and suboptimal adherence to antiretroviral therapy were recruited primarily through referrals from Los Angeles health and social service providers as well as postings on social media. Participants enrolled in the 4-week intervention received weekly counseling for adherence and daily access to web-based attention training via their personal mobile devices or computers.

**Results:**

Of the 14 participants who began the intervention, 12 (86%) completed all sessions and study procedures. Using a pretest-posttest design, findings indicate significant improvements in adherence, depressive symptoms, and attention processing. Overall, the proportion of participants reporting low adherence to antiretroviral therapy declined from 42% at baseline to 25% at intervention completion (*P*=.02, phi=0.68). Mean depressive symptoms measured by the 9 item Patient Health Questionnaire (PHQ-9) showed a substantial reduction of 36% (*P*=.002, Cohen *d*=1.2). In addition, participants’ attentional processing speeds for all types of stimuli pairings presented during attention training improved significantly (*P*=.01 and *P*=.02) and were accompanied by large effect sizes ranging from 0.78 to 1.0.

**Conclusions:**

Our findings support the feasibility of web-based attention training combined with counseling to improve antiretroviral therapy adherence among patients with psychological distress. Future research should include a larger sample, a control group, and longer-term follow-up.

## Introduction

People living with HIV are disproportionately affected by depression and posttraumatic stress disorder (PTSD) [1,2]. Compared to the general population, studies have estimated prevalence rates among people living with HIV at two to three times higher for depression and up to nine times higher for PTSD [3,4]. While both disorders adversely affect adherence to antiretroviral therapy, symptoms of depression and PTSD, even at subclinical levels, weaken an individual’s ability to effectively self-regulate the attention and cognitive processes required for consistent goal-directed behavior, such as following a long-term treatment regimen [5-7].

Research indicates that poor antiretroviral therapy adherence and engagement with HIV medical care are connected to difficulties that HIV patients face in controlling negative thoughts, memories, and impulses to effectively manage their attentional focus [8-12]. To meet the challenges associated with attaining their treatment goals, patients must learn to effectively employ cognitive self-regulatory skills, including attention control and the ability to flexibly shift attentional focus from emotionally negative health-compromising thoughts toward those that are positive or neutral and associated with favorable health outcomes [13,14].

Little research has focused on the development of interventions designed specifically to build the cognitive self-regulatory skills needed for optimal antiretroviral therapy adherence. There is, however, a growing body of empirical and theoretical evidence that demonstrates the efficacy of attention training approaches in developing these skills, thereby promoting consistent goal-directed behavior related to a wide variety of health concerns, including smoking, problem drinking, substance abuse, eating disorders, being overweight, and obesity [15-17]. Similarly, attention training procedures have demonstrated efficacy in addressing several mental health problems for both adults and adolescents, including anxiety disorders and major depression [18-21]. Such approaches provide structured training designed to strengthen an individual’s ability to shift his or her attentional focus away from stimuli that provoke thoughts and memories associated with treatment avoidance and psychological distress and toward stimuli that promote treatment engagement and emotional well-being. In addition, the indirect nature of attention training approaches makes them appropriate for addressing the implicit or nonconscious thoughts, beliefs, and memories that are related to depression, anxiety, and trauma [22,23].

Despite promising research indicating the value of attention training in addressing both psychological and physical health outcomes, there is a paucity of research focused on the application of attention training among HIV patients with comorbid psychiatric symptoms [24]. Attention training is a clinically relevant approach given that negative attention biases are closely linked to depressed mood and a tendency for threat vigilance in connection with anxiety and trauma. The training of attention away from salient negative stimuli and toward neutral or positive stimuli could be used to improve treatment adherence, increase engagement with HIV medical care, and promote mental health functioning. To enhance treatment outcomes and the durability of attention training effects, individualized stimuli could be presented during each session, thereby addressing the salient thoughts, beliefs, memories, and images that trigger specific types of behavior or emotional responses for a given patient. Research indicates that individualized stimuli trigger stronger attention biases among study participants than general stimuli [25,26].

We conducted an exploratory pilot study to develop and implement an intervention consisting of a 4-week, web-based attention training program combined with evidence-based counseling to improve antiretroviral therapy adherence and reduce psychological distress among HIV patients. Individualized, web-based attention training, in combination with evidence-based counseling, has the potential to serve as an accessible approach that could be widely disseminated to reach individuals at risk for poor treatment outcomes due to suboptimal antiretroviral therapy adherence or psychological distress. As a first step in exploring this potential role, the study sought to develop and determine whether such an intervention approach could be efficiently deployed and conveniently delivered to patient populations disproportionately affected by HIV. African American and Latino young men who have sex with men represent two vulnerable patient populations in the US that suffer from persistent elevated viremia, disproportionately high HIV transmission rates, and low levels of engagement in the HIV care continuum [27]. Given the need for effective cognitive self-regulatory skills in maintaining consistent adherence and engagement with care amid elevated psychological distress and multiple psychosocial stressors (eg, HIV stigma, experiences of trauma, childhood abuse), the study provided an opportunity to evaluate the viability of intervention featuring web-based individualized attention training to reach these vulnerable patient populations.

## Methods

### Participants

The sample consisted of participants recruited for Project STEP (Steps Toward Embodying Positivity), an intervention designed to address HIV treatment adherence and depressive symptoms among African-American and Latino young men living with HIV in the Los Angeles metropolitan area. Participants in the intervention, which combined individualized web-based attention training with evidence-based counseling, were recruited primarily through referrals from health and social service providers and through postings on social media used by the target population (eg, Adam4Adam, Craigslist, Grindr).

Individuals who expressed interest in joining the study were screened in person or by phone to determine if they met the following 4 eligibility criteria: (1) African American or Latino male living with HIV; (2) 18- 29 years old, inclusive; (3) self-identified as gay, bisexual, or same-gender loving; and (4) depressive symptoms at mild or higher levels of severity based on self-report measures or suboptimal antiretroviral therapy adherence, two psychological and behavioral risk factors for poor HIV treatment outcomes that the intervention was designed to address.

### Procedure

Upon meeting the inclusion criteria, participants were administered informed consent and then enrolled in the study. Assessments and interviews with participants were conducted by a study team member during face-to-face meetings. After completing procedures to elicit and assess their individualized stimuli (ie, brief thoughts related to treatment and mood changes), participants were scheduled for a second study meeting in which they were given an attention training tutorial and assessed for baseline reaction time performance. Upon finishing 4 weeks of attention training, participants were scheduled for a posttraining assessment within a week of their final training session. Participants received $50 each for completion of the baseline and final assessments and $5 for each weekly meeting attended. All procedures for recruitment, data collection, and confidentiality were reviewed and approved by the Institutional Review Board of Charles R. Drew University of Medicine and Science.

### Measures

#### Overview

We collected study data through use of a self-administered computerized survey that participants completed during baseline and posttraining study visits. To gauge the preliminary impact of the intervention, the computerized survey included measures of adherence and depressive symptoms. To describe our sample, we administered questionnaires tapping demographic and health-related characteristics, anxiety and trauma symptoms, and psychosocial stressors (eg, HIV stigma and childhood sexual abuse). On average, participants completed survey items within 30 minutes.

#### Demographics

Participants provided basic sociodemographic data by completing a 22-item questionnaire that requested information related to age, ethnicity, education, employment, income, HIV serostatus, healthcare usage, and other personal characteristics.

#### Adherence

Self-reported adherence was assessed using a modified version of the visual analogue scale (VAS) [28,29]. In the scale used in this study, participants were presented with a horizontal number line divided into 4 segments to represent the percentage of HIV medication doses missed during the 4 days prior to the assessment (ie, 0%-25%; 25%-50%; 50%-75%; and 75%-100%). Using the number line, participants were instructed to indicate their adherence within 1 of the 4 categories. The final category encompassed moderate and high levels of adherence. Recent research suggests that newer formulations of antiretroviral therapy may allow some patients to achieve virologic suppression and immunological benefits with relatively moderate adherence levels [30-34]. The modified version of the scale was used to enhance its administration and reduce response bias.

#### Depressive Symptoms

Depressive symptoms during the 2 weeks prior to assessment were measured using the Patient Health Questionnaire-9 (PHQ-9) [35]. The PHQ-9 is a well-validated and widely-used brief instrument for assessing and monitoring depression severity. Depression scores derived from the PHQ-9 correspond to minimal (≤4), mild (5-9), moderate (10-14), moderately severe (15-19), or severe (≥20). Based on systematic reviews and a meta-analysis of the PHQ-9, a cutoff score of 10 or greater has been described as indicative of meeting diagnostic criteria for depression [35,36]. The instrument had a Cronbach alpha of .89.

#### Trauma Symptoms

The Posttraumatic Stress Checklist-Civilian Version [37] was used to measure trauma symptoms. The checklist is a 17-item self-report measure of PTSD symptoms. Participants were asked to respond to each item using a 5-point Likert scale response format. Scores on the instrument range from 17 to 85, with higher scores indicating greater symptom severity. A score of 30 has been recommended as the minimum threshold for the further evaluation of PTSD symptoms among individuals in a civilian population [38].

#### Anxiety

The Modified Mini Screen [39] was administered to gauge anxiety symptoms. It is a 22-item scale designed to identify individuals who may have psychiatric symptoms at levels that warrant further evaluation. We used 9 items from the scale to assess anxiety symptoms among participants, with scores of 6 or greater indicating elevated levels of anxiety.

#### Childhood Sexual Abuse

Two items from the Child Sexual Abuse Index [40] were used to identify participants who had been subjected to sexual abuse during childhood. Specifically, participants were asked to indicate whether before the age of 18 they experienced (1) unwanted sexual events and/or (2) sexual abuse or molestation. The Child Sexual Abuse Index also includes additional items in which participants indicated the type of sexual abuse, whether it involved violence or physical force, their age when the abuse occurred, and their relationship to the perpetrator(s).

#### HIV Stigma

To assess the presence of HIV stigma, we used the AIDS-Related Stigma Scale [41]. Participants were asked to respond (yes/no) to 6 dichotomous items pertaining to internalized negative beliefs and perceptions about people living with HIV.

### Attention Training: Project STEP

The goal of attention training through Project STEP was twofold. First, it was designed to increase treatment adherence by teaching participants how to maintain their focus on thoughts that were approach-oriented with regard to treatment and to direct their focus away from thoughts that were avoidance-oriented. Second, to reduce depressive symptoms, attention training also sought to increase a participant’s skill in quickly diverting attention away from those thoughts perceived as having an emotionally-negative valence and directing attention toward thoughts that were perceived as neutral or having an emotionally-positive valence. Attention training, delivered via a web-based app, used the participant’s own thoughts identified during an individualized assessment procedure.

During the assessment procedure, we elicited a wide range of personal thoughts and perceptions about treatment from participants through individual interviews. Thoughts related to positive and negative changes in the participants’ emotional states were also elicited. Individual interviews were followed by administration of a computerized program in which participants were asked to quickly rate the similarity of paired combinations of their treatment-related thoughts as they appeared in random order on the computer screen. The computerized rating procedure is consistent with other research designed to identify implicit cognitive processes [42-44]. Ratings were subjected to multidimensional scaling analysis to generate 2D mappings that depicted how a participant’s treatment-related thoughts, memories, and mental associations were associated with either treatment adherence or treatment avoidance. Multidimensional scaling analysis has been used and evaluated as an approach for the assessment of implicit cognitive processes [8,43,45]. Both implicit and explicit cognitive processes were captured through the assessment procedure.

#### Modified Dot-Probe Task

Attention training was delivered through a modified version of the dot-probe task, a spatially oriented computerized procedure employed to retrain attentional focus. Using a web-based version of the task developed for the present study, participants accessed the dot-probe task via their computer or mobile device and completed sessions at home. At the start of a training session trial, participants were asked to view the screen of their device and watch a fixation cross that was situated in the center of the screen. After 1000 ms, two stimuli consisting of contrasting thoughts that were elicited during individualized assessments replaced the cross and appeared simultaneously on opposite sides of the screen for approximately 2500 ms. Then, a dot-probe appeared on the screen in the location of one of the previous stimuli. At this time, participants were required to indicate the location of the dot-probe as quickly as possible by clicking on their cursor or touching the screen of their device. The probe always appeared in the location of the stimuli that were treatment approach-oriented and conveyed a neutral or positive emotional tone, thereby training participants to respond to these types of stimuli rather than to negative and treatment avoidance-oriented stimuli.

Each individualized training session lasted approximately 15 minutes and was presented in 4 blocks. A single training block was composed of 50 trials, with a trial consisting of each sequence from the appearance of the fixation cross to the onset of the dot-probe. Completion of a training session required that the participant finish all 4 blocks. Participants, who received a tutorial practice session on the use of the attention training program prior to beginning the intervention, were provided information on the rationale behind attention training, an explanation of attention training procedures, and explicit instructions in which both speed and accuracy were emphasized. To ensure they understood how to use the computerized program, participants were required to have an accuracy rate of 80% during the tutorial practice session before proceeding to actual intervention training. During the intervention, trial-by-trial feedback in the form of an audible signal alert was provided during attention training sessions to aid participants in reorienting their attentional focus. At the end of each block of training, participants were presented with a screen that showed their reaction time and accuracy rate for that specific training session. Participants were asked to complete at least three individualized training sessions at home on a daily basis for 4 consecutive weeks. With repeated trials, participants were expected to implicitly learn how to redirect or retrain their attentional focus toward neutral, positive, and approach-oriented stimuli and away from treatment avoidance-oriented stimuli associated with negative emotional states and poor health behaviors. To evaluate changes in the amount of time a participant required to shift their attentional focus from avoidance-oriented or negative thoughts toward those that were approach-oriented or positive/neutral (ie, attentional processing speed), we used reaction time measures collected during baseline and posttraining administrations of the modified dot-probe task.

#### Weekly Counseling

In addition to attention training, participants received weekly counseling related to HIV treatment adherence and cognitive self-regulation. Two intervention counselors, who matched the age, gender, and ethnic characteristics of the sample, were trained and supervised by the principal investigator, a licensed clinical psychologist. The first of the 4 counseling sessions focused on psychoeducational content, such as the role of thoughts in health behavior and affect. Participants were given information on techniques to identify and monitor their thoughts and were encouraged to discuss how attention training could be used to effectively manage their thought processes. During the subsequent 3 meetings, participants were presented with selected modules adapted from the Treatment Advocacy Program [46-48], an evidence-based individual level counseling intervention for people living with HIV. Modules were delivered by counselors in the form of PowerPoint slides via a laptop computer or iPad. Treatment Advocacy Program modules selected for this study provided participants with behavioral strategies and information pertaining to antiretroviral therapy adherence, mood management, and alcohol and substance use. Participants were also given information on local resources and provided with referrals when needed.

### Data Analytic Strategy

Data analysis was performed using IBM SPSS 22.0. Due to the exploratory nature of the study and the corresponding small sample size, data analysis focused primarily on descriptive statistics. The full sample consisted of individuals who were enrolled into the study and completed baseline questionnaires. We examined data from the full sample (N=20) to characterize participants who met eligibility criteria and completed baseline measures. Most analyses presented in this report, however, are based on data from participants who completed the attention training intervention and final assessments (n=12). To compare participant characteristics based on study completion status, we used the Chi-square statistic for categorical variables and independent samples *t*-test for continuous variables. The Chi-square statistic was also used to examine changes in adherence among participants from pre- to posttraining assessments.

We used *t*-tests to examine changes in mean numbers of depressive symptoms, reaction times, and accuracy scores. Reaction time analyses included reaction times only from correct responses. To reduce the influence of outliers, we eliminated reaction times that were 1.5 standard deviations above or below a participant’s mean response time. This approach is consistent with other published research [49]. Effect sizes were calculated using the phi statistic for categorical variables (small effect=0.1; medium effect=0.3; large effect=0.5) and Cohen *d* for comparisons of means (small effect=0.2; medium effect=0.5; large effect=0.8) [50].The alpha level for all statistical tests was set at .05.

## Results

### Sample Description

African Americans comprised the majority of the full sample (12/20, 60%) and Latinos represented 40% (8/20). Mean participant age was 27 years (SD 1.7). Study participants identified as gay (15/20, 75%) or bisexual (5/20, 25%). Sixty-five percent of participants (13/20) indicated that they had completed high school, a high school equivalency credential, or some college, and 20% (4/20) reported graduating from college. Fifty-five percent of the full sample (11/20) had annual incomes below $20,000.

Adherence in the full sample was considerably below optimal levels, with the majority of participants (12/20, 60%) reporting adherence rates less than or equal to 75%. Participants experienced high levels of psychological distress. The mean depressive symptom score was 11.95 (SD 6.6), which is in the moderate range based on the PHQ-9 and is above the threshold widely used to suggest further evaluation for major depression. Fifty-five percent of participants in the full sample (11/20) reported elevated symptoms of anxiety. With regard to trauma symptoms, 40 percent (8/20) had symptoms at or above recommended screening level cutoffs for PTSD. Forty-five percent (9/20) reported unwanted sexual events, sexual abuse, or molestation before the age of 18. Eighty percent (16/20) reported experiencing internalized HIV stigma.

Of the 20 participants enrolled, 6 were excluded as they failed to attend a required study meeting for orientation to attention training procedures. Based on the remaining 14 participants who began attention training, the study completion rate was 86% (2 participants dropped out for unknown reasons before completing the protocol). There were no statistically significant differences between enrolled participants who completed the intervention and those who were excluded or dropped out with regard to demographics, adherence, depressive symptoms, or other assessed variables.

### Attention Training

Participants were encouraged to complete at least three attention training sessions on a daily basis, for a total of 84 sessions during the intervention. The median number of training sessions among intervention completers was 48. All 12 study participants who completed the intervention attended each of the 4 weekly meetings with a study counselor.

Table 1 shows changes in attentional processing speed among study participants. Attentional processing was based on mean reaction times scores grouped by assessment period (baseline vs posttraining) and by stimuli pairing type (ie, positive-neutral, negative-neutral, positive-negative, and neutral-neutral). Participant reaction times to correctly identify the location of the dot-probe in each of the 4 stimuli pairings significantly declined from baseline to posttraining assessments, with large Cohen *d* effect sizes ranging 0.78 to 1.0. Participants experienced the greatest mean reduction in reaction times for the positive-negative stimulus pairings (369 ms), followed by positive-neutral pairings (353 ms).

**Table 1:** Mean dot-probe reaction time to stimuli presented at baseline and posttraining assessments (n=12).

Stimuli pairing	Baseline mean reaction time, ms (SD)	Posttraining mean reaction time, ms (SD)	Test statistic	*P* value
Positive-neutral	2553 (693)	2200 (375)	–3.25	.01
Negative-neutral	2579 (770)	2255 (414)	–2.71	.02
Positive-negative	2561 (677)	2192 (368)	–3.48	.01
Neutral-neutral	2483 (683)	2175 (331)	–3.23	.01

Accuracy scores for each of the 4 types of stimuli pairings during the baseline and posttraining assessments were calculated to provide a measure of the rate at which participants correctly responded when prompted to indicate the location of the dot-probe. The only statistically significant change in accuracy scores from baseline to posttraining assessments, however, occurred when participants responded to positive-negative stimuli pairings (94% vs 98%, respectively; *t*_10_=4.05, *P*=.002).

### Treatment Adherence

We examined the relationship between attention training and antiretroviral therapy adherence before and after the intervention by examining participant adherence rates based on a threshold of 75%. We categorized participants with adherence at or below this threshold as having “low” adherence and those with adherence above the threshold as having “moderate/high” adherence. In the current study, participants with low adherence reported rates between 50% to 75%. At baseline, 42% (5/12) of participants who completed the intervention had low adherence versus 58% (7/12) with moderate/high adherence. During the posttraining assessment, however, these percentages had shifted significantly with 75% (9/12) reporting adherence in the moderate/high range (Table 2). This change in adherence represented a large effect size (phi coefficient=.68).

**Table 2:** Changes in antiretroviral therapy adherence and number of depressive symptoms among intervention participants (n=12).

Characteristic		Baseline	Posttraining	Test statistic	*P* value
**Adherence rate, n (%)**				5.6	.02
	≤75%	5 (42)	3 (25)	N/A^a^	N/A
	>75%	7 (58)	9 (75)	N/A	N/A
Depressive symptoms, mean (SD)		13.4 (6.8)	8.6 (7.5)	3.71	.002
**Symptom severity, n (%)**					
	Minimal (0-4)	1 (8)	3 (25)	N/A	N/A
	Mild (5-9)	3 (25)	6 (50)	N/A	N/A
	Moderate (10-14)	2 (17)	0 (0)	N/A	N/A
	Moderately-severe (15-19)	4 (33)	2 (17)	N/A	N/A
	Severe (20-27)	2 (17)	1 (8)	N/A	N/A

^a^N/A: not applicable.

### Depressive Symptoms

Mean depressive symptoms among participants who completed the intervention declined significantly by 36% based on the PHQ-9, from 13.4 (SD 6.8) at baseline to 8.6 (SD 7.5) posttraining, (*t*_11_= 4.16, *P*=.002; Cohen *d*=1.2, indicating a large effect size). The mean baseline score, which was in the moderate range with regard to depressive symptom severity, exceeded the cutoff of 10 widely used to suggest further diagnostic evaluation for major depression. The mean posttraining score, which fell below this cutoff, was clinically significant in that it represented an overall downward shift in symptom severity from the moderate to the mild range. As Table 2 shows, compared to baseline levels, there was a marked drop in the percentages of participants who experienced depressive symptoms in the moderate, moderate-severe, and severe ranges upon completing the study.

## Discussion

### Principal Findings

In this exploratory pilot study, we aimed to develop and implement an intervention consisting of individualized, web-based attention training combined with evidence-based counseling to promote adherence to antiretroviral therapy and reduce depressive symptoms among HIV patients experiencing elevated levels of psychological distress. Findings indicate that web-based attention training in addition to counseling can be efficiently deployed and conveniently delivered to a vulnerable HIV patient population with suboptimal antiretroviral therapy adherence and disproportionately high rates of depressive and PTSD symptoms. The intervention implemented in this study had a high completion rate (12/14, 86%), indicating strong viability as a clinical approach. Participants were able to access an individualized attention training program through their own mobile devices or computers, completing a median of 48 sessions during the 4-week attention training program, or the equivalent of approximately two sessions per day. Attention training combined with evidence-based counseling yielded considerable therapeutic benefits to intervention participants. We found statistically significant improvements among participants in antiretroviral therapy adherence from pre- to posttraining. In addition, we found both statistically and clinically significant reductions in depressive symptoms. Findings also showed notable improvements in attentional processing speed based on reaction time measures. Research suggests that improvements in processing speed play an important role in promoting everyday functioning and quality of life [51,52].

While participants in this study were assessed at only two time intervals, the study effectively employed strategies that could be used to maximize the benefits of attention training and strengthen the long-term durability of intervention outcomes. Our intervention used specific strategies (eg, performance feedback) to enhance the learning experience of study participants, drawing from recent research involving attention training [53-62]. Based on goal setting theory [63], these strategies included providing participants with explicit instructions, a clear statement of the training goal, and trial-by-trial feedback on performance (eg, reaction time changes; response accuracy rate). This study illustrates how individualized, web-based attention training for HIV patients with psychological distress could be employed in combination with psychotherapy.

Participants represented individuals who could most benefit from attention training due to the cognitive burden posed by multiple psychosocial stressors (eg, clinically-significant depressive symptoms, experiences of trauma and abuse, and internalized HIV stigma). Future studies, however, should be based on larger samples that include women and individuals representing a wider range of ages, geographic locations, and behavioral risk groups. Although studies support the validity of self-report measures of adherence [64], findings in this investigation could be bolstered by future research that incorporates biomedical measures of adherence. In addition, future studies should be designed to examine measures of cognitive self-regulation related to attention control, cognitive flexibility, and attention bias. Such measures would be derived based on administration of a standard dot-probe task where all stimuli are targeted with equal probability. This preliminary pilot study did not administer the standard dot-probe task. Finally, to better understand the role of attention training in HIV patient outcomes, research should be conducted that tracks participants over longer time intervals with a design that incorporates other approaches and a control group.

Attention training has shown much promise as an approach to improve outcomes associated with a range of health behaviors and psychological disorders [15-21,24,65]. This exploratory study contributes to the literature on attention training by showing its clinical applications in addressing the impact of depressive and trauma symptoms on HIV treatment adherence. We were able to provide evidence of the ability of individualized, web-based attention training to yield favorable improvements in adherence and psychological distress in two vulnerable populations of HIV patients. Our findings provide support for additional exploration of this promising application.

### Data Availability Statement

The raw data supporting the conclusions of this manuscript will be made available by the authors, without undue reservation, to any qualified researcher.

## Abbreviations

PHQ: Patient Health Questionnaire
PTSD: posttraumatic stress disorder
